# Enabling factors for specialist outreach in western KwaZulu-Natal

**DOI:** 10.4102/phcfm.v10i1.1690

**Published:** 2018-05-17

**Authors:** Robert I. Caldwell, Merridy Grant, Bernhard Gaede, Colleen Aldous

**Affiliations:** 1 School of Clinical Medicine, University of KwaZulu-Natal, South Africa; 2 School of Nursing and Public Health, University of KwaZulu-Natal, South Africa

## Abstract

**Background:**

There exists a major disparity in access to specialist care between patients in urban and rural areas. Specialists are a scarce resource and are concentrated in urban areas. Specialist outreach attempts to fill the gap in service provision for patients situated remotely. While there is international evidence that multifaceted specialist outreach has achieved varying levels of success, factors that influence the effectiveness of outreach have not yet been fully elucidated in South Africa.

**Aim:**

This study attempts to uncover some of the factors that enable good multifaceted specialist outreach.

**Setting:**

The study was conducted in hospitals in western KwaZulu-Natal province. This health area is served by a tertiary hospital and 20 peripheral hospitals; three of these are regional level and the majority are district level hospitals. Specialist outreach emanates from the tertiary hospital.

**Methods:**

Specialists providing outreach services from the tertiary hospital and medical officers at seven receiving hospitals were interviewed to explore perceptions regarding factors that might enable successful specialist outreach. Framework analysis on the transcribed interviews was carried out using NVivo version 11.

**Results:**

A major positive finding concerns the relationships formed between outreach specialists and doctors at the recipient hospitals. The management of the programme with respect to structure, dependability, data management, transport provision, communication technology and public health systems was also seen as beneficial in specialist outreach.

**Conclusion:**

Specialist outreach plays an essential role in providing equality in health care. To enable effectiveness, it is important to make full use of the multifaceted nature of this intervention.

## Introduction

South Africa (SA) and KwaZulu-Natal (KZN) are not unique regarding the inequality of specialist services available to different sections of the population: this is a worldwide phenomenon. Nevertheless, this distinction could not be emphasised more starkly than by that between urban and rural citizens of western KZN.

The situation has changed little since Dr Henry Gluckman’s comment at the time of SA’s National Health Commission in 1942 (exactly 75 years ago): ‘Where the need is greatest the supply of hospitals is least’, referring to impoverished remote rural areas; nor since Tudor-Hart’s 1971 *inverse care law* which stated, ‘availability of good medical care is inversely related to the needs of the population’.^1,2^

The health care system in SA is based on the district health system, which is essentially a hierarchical model.^3,4^ There are tertiary hospitals, which offer specialist and super-specialist services, and regional hospitals, which offer general specialist services. The district hospitals are run by medical officers (MOs) who are generalists, although occasionally there may be a doctor qualified in family medicine in the staff. In KZN, however, this category of hospitals is usually staffed by generalist MOs.

A feature of the health care system in KZN is that some form of specialist outreach (SO) services has been in place for many years.^5^ At first, it was mainly volunteer-based, with specialists from a few disciplines visiting outlying hospitals on an ad hoc or more regular basis. The outreach service was formalised in the late 1990s with the introduction of a dedicated transport system and a more structured visit, and from 2007, specific SO posts in all the major disciplines at principal specialist level were created in Health Area 2, or western KZN.^6^ A key feature of the SO was that it included progressively a range of activities such as consulting booked outpatients, participating in clinical governance, doing ‘problem’ ward rounds and giving a continuing medical education session. In some disciplines, it included performing operations or giving anaesthetics, reviewing equipment needs or even participating in interview panels. Such outreach has been termed multifaceted specialist outreach (MSO) and has been employed internationally and in KZN as a means of improving access to specialist services.^5,7,8,9,10,11,12,13^

The routine data collected as part of the outreach visits have provided the opportunity for quantitative analysis of output (e.g. the number of hospital visits, patients consulted and personnel contacted and diagnostic categories are recorded).^10^ However, little has been documented regarding the nature of the relationships established between the specialists practising outreach and the doctors in the periphery and what enables such encounters.^5,10^ This article attempts to describe aspects of these relationships and other enabling factors which may render outreach services effectively.

## Research methods

### Study design

The study used an exploratory qualitative research design to access rich accounts of participants’ experiences and perceptions of SO.^14^

### Setting

**FIGURE 1:**
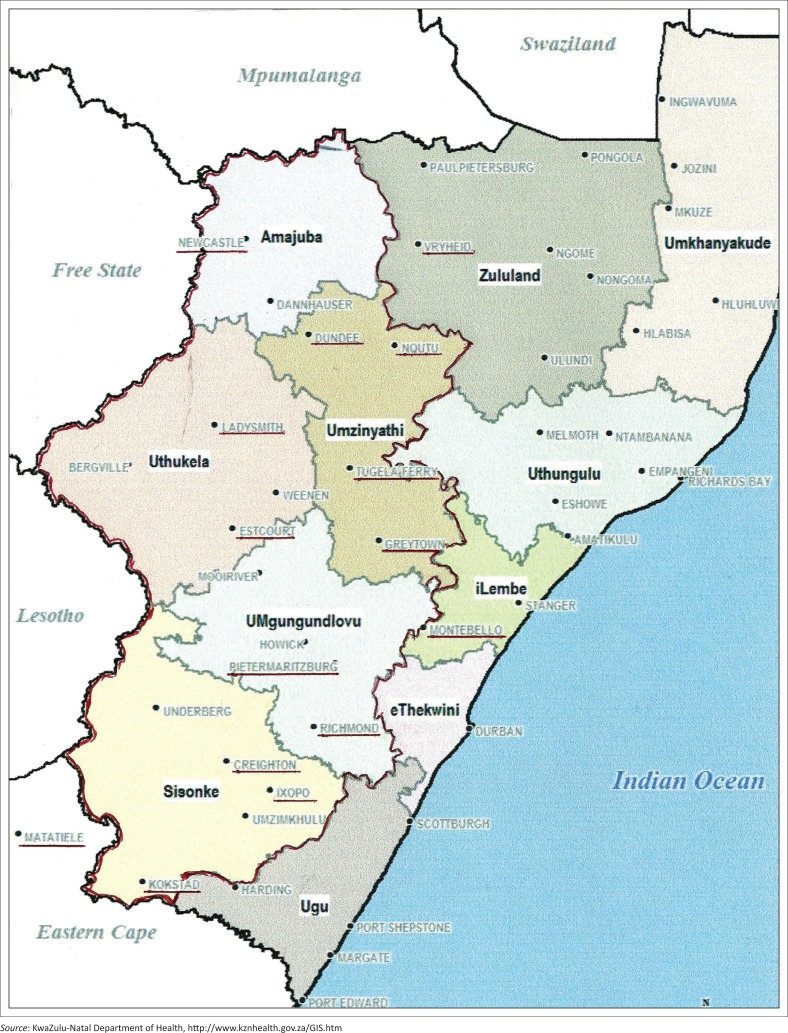
Map of KwaZulu-Natal showing the catchment area of Grey’s Hospital.

Health Area 2 (as it was prior to recent slight geographical rearrangement) occupies most of the western half of the province of KZN and serves a population of more than 3 million. Pietermaritzburg (PMB) is the urban hub and provides specialists in all major disciplines from tertiary level Grey’s Hospital and regional level Edendale Hospital. There are 20 hospitals in the health area, mostly peripheral at district level (three at regional level), half of them in rural settings, the remainder situated in small to medium-sized towns (Figure 1).

### Study population and sampling strategy

Participants were specialists from PMB and MOs based at seven of the 20 peripheral hospitals. The peripheral hospitals were specifically selected based on their exposure to regular MSO. One of the authors (R.I.C.) had also visited the seven hospitals regularly when he was outreach physician for internal medicine, and there was an established relationship with these sites.

Specialists and MOs were purposively selected for inclusion in the interviews based on their previous and current involvement in MSO. The specialists were selected to represent the clinical departments involved in regular MSO: internal medicine (two), paediatrics (two), general surgery, obstetrics and gynaecology (O&G), anaesthetics, orthopaedic surgery and psychiatry. Most of them were responsible for the organisation of MSO within their department. The participants at the peripheral hospitals included the medical manager or experienced MOs who were involved with MSO.

### Data collection

The interviews took place between August and November 2016. They were conducted by the lead author (R.I.C.) and were arranged in advance. Interviews with specialists were conducted in the consultant’s office in the central hospitals and interviews with the MOs and medical managers took place in a private room in the relevant peripheral hospital.

Although there were separate interview guides, the majority of questions were common to both groups, in particular the key questions (Box 1). The full interview was audio-recorded on the interviewer’s mobile device.

Box 1 Summary: Key questions for participants, specialist and peripheral.

**Table:** 

KEY QUESTIONS FOR PARTICIPANTS
Is multifaceted specialist outreach output recorded, and is there measure of outcomes? Are multifaceted specialist outreach activities structured or adaptable? Does multifaceted specialist outreach quality depend on the hospital visited? Does multifaceted specialist outreach quality depend on the visiting department? Is liaison important? Does the effectiveness of multifaceted specialist outreach outweigh the shortcomings? Could there be improvements in transport? Is telemetry of use? Are there better means of equitable access to specialist care? Should multifaceted specialist outreach be part of the national health insurance?

The interviews were saved and stored securely on a disc and were transcribed verbatim by a professional agency. To maintain the anonymity of participants, the transcripts were given pseudonyms: S1 to S9 for specialist interviews and P1 to P14 for peripheral interviews.

### Data analysis

Framework analysis was the primary analytic strategy whereby both a *priori* and inductive themes were identified.^15,16^ Transcripts were entered into qualitative data analysis software (NVivo version 11). Interview data were coded by all of the authors and analysed using the five stages of thematic framework analysis which included familiarising, identifying a thematic framework, systematically applying the framework to the data (indexing), creating a summarised matrix for each theme (charting) and interpreting.^15,16^ The detailed analysis was primarily performed by R.I.C. and M.G., and all authors met regularly to discuss theme development and to resolve any interpretation discrepancies in the analysis process.

Validity was sought through the consistency of findings and accurate representation, by checking for aberrant cases and through the use of original data, for example using quotations from many interviews rather than concentrating on one source.^14^ The researchers engaged in a process of reflexivity throughout the study. The primary researcher in particular reflected on the role as a researcher and outreach specialist, and this position was acknowledged in the analysis process.^14^

### Ethical considerations

Prior ethical approval for the interviews was gained from the Biomedical Research Ethics Committee at the University of KwaZulu-Natal (reference: BCA430/14) and the KwaZulu-Natal Department of Health (KZN DOH), (HRKM ref 303/17; NHRD ref: KZ_2016RP55_103) as well as from the hospitals concerned. Written informed consent was obtained from the participants before each interview.

## Results

In-depth interviews were conducted with 23 doctors: 9 specialists in PMB and 14 MOs or medical managers at peripheral hospitals who had participated in the outreach programme.

Addressing the factors enabling effective MSO, the dominant themes emerging from the analysis included the significance of relationships or liaison in MSO, structure, the importance of regular outreach, documentation, transport, technology and public health strategies (Table 1).

**TABLE 1:** Consolidated nodes and dominant themes.

Dominant theme	Consolidated nodes
1. Relationships	Liaison (in-reach)
2. Posts, departments, structure	Dedicated posts for outreach, departments, filled posts and structure of visit (booked outpatients, protocols, teaching, ward rounds)
3. Regular effective outreach	Adaptation of programme to needs, alternatives to outreach, effective outreach, factors influencing quality of visit (providing hospital, receiving hospital), giving regular outreach (structure), length and frequency of visit, needs (needs of provider hospital, needs of recipient hospital), responsibilities and regular outreach
4. Documentation	Outcome (evidence of benefit), output and record-keeping
5. Transport	Transport (air, road)
6. Technology	Videoconferencing (educational, telemedicine)
7. Public health strategies	District clinical specialist teams (DCST) and National Health Insurance (NHI)

### Relationships

To the question: ‘Is liaison or the building up of relationships with personnel at recipient hospitals important to the functionality of the programme?’, the answer was a unanimous ‘yes’, for both central and peripheral interviews, and in most cases emphatically so, with words like ‘the most important benefit’ and ‘absolutely’ recurring. Several participants stressed that liaison was likely to be more important than the actual clinical performance offered by the specialist during the outreach visit.

‘… probably the most important component … everybody thinks outreach is by definition access to health care to patients … it’s not really that. That’s what happens when you get it right … it’s actually building … working relationships and up-skilling of peripheral hospitals.’ (S9, female, specialist)

Both specialist and peripheral participants reported that such relationships could save patient lives.

‘… that’s the most important function, just establishing that relationship … [*it*] allows so many patients to be saved literally because you’ll find that most doctors in that hospital will now say: “okay, who’s the outreach”, “okay let me phone him because there was some miscommunication with maybe a registrar or a medical officer here” in terms of accepting the patient and they will phone that outreach doctor and use that outreach doctor to actually either get advice, proper advice, or get the patient transferred.’ (S1, male, specialist)

A similar comment noted that the relationship facilitated effective decision-making and the taking of responsibility across different levels of care, and facilitated access to scarce health care resources:

‘But having a specialist that you can phone and say, “Hey doc, you know please help us with this guy”, we can save a life here, we can really make a difference, can you help us facilitate something like a dialysis or an Echo or radiotherapy …’ (P2, male, MO)

It reflected a shared commitment and responsibility by both specialists and MOs working in the periphery, and recognised the possibility and importance of working in effective teams across the levels of the health care system.

‘…we really tried to work very hard at making the staff out in our district and regional hospitals feel that they are all part of the same team and that the patients that they are caring for out there are as much our responsibilities as they are theirs. So that when they phone us for whatever reason, we are not sharing them as a favour….’ (S6, male, specialist)

One participant from a peripheral hospital (P3) reported that the fact that consultants are prepared to travel great distances to visit the hospitals is indicative of their commitment, motivation and enthusiasm to their work in outreach. This reflected an aspect of how the relationship between the specialist services and the peripheral district hospitals could be built.

The importance of communication was stressed, and participants made it clear that it was the establishment of a relationship, even a friendship, that made it possible for worthwhile communication to continue and thrive.

‘… when we met people and they come and had cake and tea with us – even if they didn’t teach us a single thing – it’s that interaction that I think strengthens the relationship. When you phone you’re a face and … you know they understand you more and they are more likely to help you ….’ (P1, female, MO)

The place for up-skilling was quoted. Again, opinions indicated the limitations of clinical benefit provided on a particular visit: it was the potential for the up-skilling of the recipient staff that might result in good outcomes.

‘… a main responsibility for outreach is to up-skill the staff that are working at the recipient hospitals … there’s only so much you can do by seeing individual patients. If you up-skill, you can make a much bigger difference to the quality of … health services provided at that hospital.’ (S8, female, specialist)

In-reach, the reciprocal or the corollary of outreach, is commented on. Telephone communication between doctors who have an established liaison is the simplest form of in-reach.

‘… it’s also partly because I go ahead and encourage them to phone me if they have a problem. It’s almost a continual electronic round going on ….’ (S6, male, specialist)

The limitations of more sophisticated in-reach, for example where MOs from peripheral hospitals spend time at the specialist hospital, received cautioning.

‘… in-reach has got a role to play and this is where the doctors from the district actually come to … the tertiary hospital, but that programme has to be thought through very carefully to make sure that that two weeks spent there is maximum benefit because during the time when they are away … then the base-hospital is short of doctors.’ (P9, male, MO)

A participant (S4) commented on the difficulty that the management of peripheral hospitals might have in relinquishing staff even for short durations of in-reach:

‘Management of these centres are unable to release their staff for in-reach. They’ll say look, if I send this gentleman to you for a week, who’s going to run my casualty?’ (S4, male, specialist)

### Structure of the outreach programme

There was agreement from both the specialists and the MOs in the periphery that a structured MSO programme was officially in existence. However, there were comments about the unavailability of key personnel. Specialists interviewed were of the opinion that their department deserved the appointment of an outreach specialist, for whatever role that department used such a post.

‘… our department does not have a consultant head of unit post at all [*laughing*]. Never mind one dedicated to outreach. So, we’re currently motivating for a consultant head of unit post ….’ (S8, female, specialist)

Most specialist departments had been allocated a formal funded outreach post a few years previously, but not all such posts were still existent. Some departments no longer used the post exclusively for outreach: the trend was to spread MSO responsibilities within the specialty.

‘… the other clinicians weren’t keen on doing outreach because they saw it as being the responsibility of the person who’s employed purely for outreach, and that made it more difficult to convince them that they were equally responsible for outreach. So I think having a dedicated individual can be an obstacle in the long run ….’ (S1, male, specialist)

While establishing their outreach programmes, some departments developed a uniform structure that was expected to be followed as part of their MSO visits. The facets that should be covered during an outreach visit were outlined clearly.

‘…although it’s called the KwaZulu-Natal [*X department*] outreach programme it was actually developed here in our own department, so our department’s methodology and structure has become a provincial guide really.’ (S6, male, specialist)

Other departments tried for flexibility: the longer MSO was provided, the more it became apparent that different recipient hospitals required adaptability and flexibility. A rigid and overstructured approach might not serve the needs of every hospital.

‘That’s something that I haven’t cleared up completely in my mind. Each hospital is very different, strikingly so and so I certainly do adapt to the hospital that I’m going to, but … we have to abide by some fixed activities … and quite how to keep the standards but retain the flexibility, I haven’t got a nice, clear answer for that yet.’ (S3, male, specialist)

The MSO visits consisted of combinations of ward rounds, outpatient clinics and procedures in theatre and teaching, and most peripheral hospitals regarded such visits as worthwhile. The clinical structuring of a visit was predictable in view of limited options, but again the need for a particular component differed amongst hospitals: for example, one might require a large booked outpatient clinic, at another there might seldom be the need to see outpatients.^9,10^

‘[*We would*]… start off with ward rounds, problem rounds … [*which*] take about two hours and during that period the visiting consultant does do bedside teaching and we have medical officers that join the round… Thereafter we proceed to the out-patient clinic … have on average about twenty patients booked …’ (P10, male, MO)

The arrangements were not always mutually satisfactory, and a focus on clinical work only was of limited value. This pointed to the advantage of the multiple facets of the outreach visit, rather than its being merely a specialist clinical service.

‘And then our [*specialty Y*] usually hurtles in at about half past eight and is out of here by about ten and we are not 100% sure [*laughs*] if there are benefits on it. Then we would see some patients in the ward – very few though and … a handful of outpatients… usually not, a teaching session ….’ (P1, female, MO)

### Regularity of outreach

Peripheral hospital participant MOs made it clear that, despite the intentions of specialist departments, their hospital did not receive outreach from every such department. This was regarded as a significant shortcoming.

‘There are inconsistencies and we would very much appreciate [*specialty Z*] to do visits here to understand the pressure that we are up against ….’ (P2, male, MO)

The necessity for making maximum use of the visit was emphasised by both the visiting specialists and the hosting MOs. While travelling time was acknowledged as a burden, this could be offset by the efficiency of a well-planned process once the visitor was on site.

‘If the day is well structured you can use the time well … some of the hospitals are far away so there is more time travelling and less time for … work. But you have to use that time well. There is no way around that really.’ (S6, male, specialist)

Both groups stressed that MSO should be regular with an appropriate frequency (S9). The reliability of an ongoing and continuous commitment was a critical requirement even if the correct frequency was still to be determined: sporadic erratic visits were unacceptable (S9) and undermined trust in the outreach service. Cancellation of a visit was distressing to both patients and recipient doctors.

‘People think that if you do outreach three times a year, it must be three perfect visits. That’s the only way outreach must work. [*but*] … you should probably do outreach 12 times a year … have two brilliant visits and two terrible visits and the rest of them are going to be mediocre or okay and average, but on balance you’re winning. It’s the slow plodding steps.’ (S9, female, specialist)

‘… the most important thing is there needs to be continuity. There is nothing as bad as, that there be booked patients and they [*the specialists*] don’t come.’ (P13, male, MO)

While a monthly visit was regarded as the standard in terms of frequency of visits, this was by no means the uniform choice – fortnightly or even weekly visits were suggested. There was also the possibility of reduction in the frequency of visits once a programme was fully up and running. Similarly, visits lasting more than a day were regarded as potentially useful, particularly in some surgical disciplines where procedures could be planned and performed and post-operative review and care could be offered. However, there were limitations and recipient hospitals had to appreciate that visiting specialists also had vital obligations to their own central hospital.

### Documentation

It was recognised that the collected data could be a major facilitator for the MSO project, not only in terms of generating a record of the process but also in assisting with the evaluation and planning of the service.

‘… the former outreach specialist in our department did a sterling job of gathering tons of data to the point of having … publication[*s*] out of all of his work… I want to actually build an outreach database for the department. The one thing that was lacking to me is institutional knowledge…’ (S9, female, specialist)

However, the data collection depended on clinicians ensuring that every visit was documented in a structured and coherent manner. This was not always the case, as participants commented:

‘The doctors are not very good at sending their reports, so there are a lot of gaps.’ (S2, female, specialist)

Measuring outcomes, evidence of the benefit provided by MSO, was acknowledged as being challenging – although there were some supportive data. There was approval of the seeking of opinions of doctors involved in providing or receiving MSO.

‘… measuring outcomes is terribly hard. … the outputs that would be most useful to measure would be doctors’ opinions and doctors’ sentiments of those places in receipt of outreach …’ (S3, male, specialist)

It was evident that the current routinely collected data did not give a comprehensive view of the service. While the benefit to patients of gaining faster access to specialist care with less travelling was acknowledged, it was clear that the system-wide benefits were difficult to measure.

‘… offering the population here … much quicker access to specialist care where it might have taken them very long to see a specialist … obviously they quite happy to see a specialist in [*Hospital Z*] rather than Grey’s Hospital because it prevents them from travelling … that’s more or less what benefit we derived from it.’ (P10, male, MO)

### Transport

The existing transport arrangements by the Red Cross Air Mercy Service (AMS), contracted to provide a light aircraft and road travel service to many hospitals throughout the province, were appreciated.^6^ While both forms of transport had some obvious drawbacks, like bad weather and dangerous roads and driving (S1, S9), there was broad acceptance that a transport system was essential for the mere existence of a structured and regular MSO in a province like KZN.^9,17^

‘The transport arrangements are pretty good … The weather can be a problem sometimes, but you still need to fly if you’re going to a very far place… the only way to get there. If you drive two and a half hours to somewhere like [*X*] and to drive … back, it’s not really beneficial unless you’re going to stay there overnight, which I don’t think many of our clinicians are keen to do.’ (S1, male, specialist)

There was concern that there had been delay and uncertainty regarding the renewal of the AMS contract, with strong support for the existing transport provider. There was also reassurance that the MSO programme would survive a change, and there was an opinion expressed that the DOH itself should be the provider.

‘… (MSO) would suffer greatly if AMS were not to get the contract, unless it were replaced with something that works in similar… – but I don’t think it would fall apart, I think we just have to find an alternative method of transportation.’ (S2, female, specialist)

Whatever the arrangement, it was emphasised frequently that a coherent and well-coordinated transport system for specialist visits helped to enable effective outreach services.

### Communication technology

Telehealth was regarded as valueless unless it functioned properly, with adequate maintenance and updating of equipment.^18^ Numerous comments bewailed dismal experience with the existing official videoconferencing systems at both peripheral and central hospitals, even if, as a concept, telehealth might be particularly relevant to a department (S8).

‘Telemedicine is not a good medium for, for anything actually. The technology does not seem to work often… it requires careful management … you can’t just dial in and [*we do*] not know some of the basic skills that are needed in order for the communication to be effective.’ (S6, male, specialist)

While there was support for this view from some participants, other innovative telecommunication systems have been used more frequently.

‘Because we need to invest in telemedicine …by doing that, we will be able to do tele-conferencing, we will be able to discuss, will be able to get visual support, will be able to understand … give the clear picture to the consultant then and there… the other thing … is the WhatsApp system.^19^ It is working wonders….’ (P6, female, MO)

‘We’re not using [*telehealth*] at the moment. It may have a role. We are partially using sort of WhatsApp, some sort of tele-medicine for … outreach … So it’s something new that we’ve been implementing now over the past two years….’ (S1, male, specialist)

‘The official one is not working at all at the moment. The unofficial one, which is WhatsApp, is definitely used and it’s quite good. ECGs, pictures, videos, very good….’ (S3, male, specialist)

The availability of adequate telecommunications, including appropriate speed for data connections, was recognised as a limitation at a number of remote sites. Moreover, it was emphasised that videoconferencing should enhance but by no means replace MSO.

‘They were trying to get our doctors there to use more of the tele-medicine, but we don’t want to miss that face-to-face interaction. Because like you were saying with the liaison, that’s where it is built, and doing that across tele-medicine you can’t do. So … I don’t want to replace what we’re doing, only add to it, if that makes sense.’ (S2, female, specialist)

There was strong support for a specific telemedicine pilot intensive care unit (ICU) link up, which was proving very successful.

‘We used to link up for the Grey’s Wednesday morning meetings previously and that has now ceased but we do have the ICU one going on at the moment and with good results I must add.’ (P10, male, MO)

### Public health strategies: Aligning with National Health Insurance

The most prominent public health intervention in SA is the National Health Insurance (NHI), which is intended to be fully established within the next decade. There was strong support for MSOs being an integral part of the NHI, and agreement that its omission from the White Paper was a serious flaw.^20^

‘NHI is about getting affordable health care to everybody. Outreach is about getting access to everybody, same thing in a sense … the intent is the same. We’re trying to get health care to people who need it out there.’ (S9, female, specialist)

‘If you don’t know what’s out in the catchment area, how can you run your service properly? You’ve got to go out, you’ve got to go and see what’s out there: and what is out there, changes. So you’ve got to carry on going out.’ (S3, male, specialist)

A strategy which is included in the NHI White Paper is to base a range of specialists, confined to obstetrics, paediatrics and anaesthetics together with family medicine, at the district level. This district clinical specialist team (DCST) concept came in for much criticism by participants both centrally and in the periphery.^21^ Although the teams were regarded as complementary to MSO clinicians according to the comments made by participants S2 and S6, the majority of those familiar with the concept, even those in specialties included in the teams, felt that the national DOH was on the wrong track, risking duplication of scarce resources (S1, S2, P4, P9). A particular criticism was that these teams were district-based and therefore office-based, instead of being clinically involved (P1, P6, P8, S4).

‘… very short-sighted when they introduced DCST … unfortunately… only covers … a small proportion of the actual population … disciplines who probably already had resources in place…It should be one outreach programme … coordinated centrally…’ (S1, male, specialist)

‘Doesn’t work as well as specific outreach; because they are so busy doing stuff at the district office … they certainly don’t come and … do clinical training or up-skilling or ward rounds … I think it’s a fun idea … for us it doesn’t have a direct benefit.’ (P1, female, MO)

## Discussion

Concerning the importance of liaison or relationships in outreach programmes, participants, whether they were specialists providing MSO or MOs at peripheral hospitals receiving MSO, had the same opinion. Therefore, SO, even if multifaceted, need not be viewed as simply bringing services and teaching to the periphery: liaison may be more important than the actual clinical performance of MSO. ‘Outreach’ may even be a patronising and dated term and should perhaps be replaced by another: ‘liaison’ or ‘relationships’ or ‘collaboration’. Indeed, collaboration may capture the essence of MSO as it was regarded as its crux by so many participants in this study. Teamwork and shared responsibility were also identified as important elements of outreach. Therefore, the collaboration component extends to a joint responsibility for the recipient hospital as a whole.^22,23^

In-reach is a consequence of outreach and may be regarded as its reciprocal or corollary. A call to the centre need not be merely a telephonic one. If it involves a known specialist who encourages such communication in order to facilitate or obviate a referral, then that call can be regarded as the simplest form of in-reach. This can become increasingly sophisticated, culminating in the opportunity for doctors at the peripheral hospital to visit the specialist hospital for the purpose of ‘up-skilling’ – either for a short time, a day, or for an attachment of, say, 4 weeks, to learn anaesthetics.

However, although a vital accompaniment to outreach, in-reach is anything but a ‘one-fits-all’ solution. Its planning has to be careful, so that the downside of staff shortage at the peripheral hospital does not outweigh the benefit of the up-skilling to be gained at the specialist central hospital. The medical literature reveals a paucity of research on the in-reach component of SO, a gap which needs to be filled.

The establishment of a sustainable MSO programme is clearly dependent on a long-term relationship or liaison between the specialist hospitals and the recipient peripheral hospitals, and this is well supported in both the international and South African literature.^12,24^

A formally structured multifaceted visit arranged in advance may enable effective SO. Area 2 MSO, however, has evolved into allowing departments and recipient hospitals to organise the programme in the manner most appropriate to and efficient for its needs and desired achievements. This flexibility of structure becomes an advantage, provided that there is sufficient consistency to maintain a standard, within the minimum requirements set by particular departments. This aspect, flexibility according to needs, may contradict the literature which recommends increased structure in order to improve visits.^25^ However, some consultants were reluctant to contribute to MSO, as confirmed by the interviewer’s experience as outreach specialist in a department: this may detract from an effective structure.

Regularity of MSO – and perhaps more importantly, that the consultant can be trusted to visit rather than offering the uncertainty of an ad hoc arrangement – is also an important enabling factor towards the building of a more substantial relationship and thereby better outreach. There was unanimous confirmation that to be effective and sustainable, MSO should be performed in a reliable regular manner with an appropriate frequency. However, although monthly is the current practice, there was a strong opinion towards more frequent visits, particularly if the NHI is to become reality.

As a result of the outreach service, considerable data have been collected by the visiting specialists as a requirement of their departmental duties, as well as by AMS, which was contracted in 1998 by the KZN DOH to provide both air and road transport for the MSO to and from most of the peripheral sites.^6^ Documentation implies accurate record-keeping and analysis of the data thus acquired, and the interviews revealed considerable shortcomings in both aspects and emphasised the difficulty in assessing outcomes. However, the interviews also confirmed that the opinions of participants were important in measuring outcomes or benefits. This support for interviews as a means of assessment is unexpected and reassuring.

Although a great deal of data are potentially available, they were seldom used to full advantage, and there is a need for prospective studies, particularly in the estimation of outcomes or benefit to the patient and recipient hospital and its medical staff. The data are readily available for the taking, and the literature, international literature in particular, illustrates the value of such documentation in assessing the efficacy of SO.^25,26,27^ Moreover, the failure of adequate records and analyses points to the relatively low calibre of the management of the outreach service. This may limit planning and jeopardise the resourcing and sustainability of the MSO services.^24^

Outreach by its very definition demands transport:

… any type of health service that mobilises health workers to provide services to the population or to other health workers, away from the location where they usually work and live…^28^

Nevertheless, the need for an efficient arms-length provider becomes even more vital when the area’s geography dictates this, as in western KZN, where AMS provides both air and road transport. The former is essential when huge distances are to be travelled, to-and-fro, in the course of a day. The latter allows access to hospitals which are too far away from airstrips to justify flying, or close enough to the centre to make road travel more logical.^6,9^ A rule of thumb might be that trips lasting less than 2 h should be by road and longer trips should be by air provided that there is a suitable landing strip for that hospital. Motor vehicle accidents are, however, notoriously prevalent in SA, and even with flights, road transport is required between landing strips and hospitals.^17^

The importance of an independent efficient transport system was strongly supported, and praised, by air and road. Opinions reflected the importance of road travel and emphasised that it should not suffer Cinderella status compared to air travel. There was concern that AMS might not regain its contract. The agreement between KZN DOH and AMS in 1998 put MSO in KZN onto a formal footing and has been an undoubted enabling factor in its sustainability since then.^5,6,9,10^ Conversely, if there were no competent transport provider, MSO services could not survive.

Telehealth facilities, using videoconferencing to provide opportunity for tele-education and telemedicine, have been set up in a number of central and peripheral hospitals in SA and KZN. Theoretically, these should enhance MSO by facilitating greater links between the consultants and doctors in the peripheral hospitals, and could therefore be considered an important tool. Telehealth may be used as a generic term for the provision of health care remotely by means of telecommunications technology.^18^ The use of telehealth, videoconferencing for tele-education or telemedicine (the clinical application of telehealth) is well described in the rapidly burgeoning international literature as being of value in MSO.^29,30,31,32^ Although telehealth should enable MSO but not replace it, there was stern criticism of the inadequacies of the technology employed. This is corroborated by the experience of an author (R.I.C.) during involvement in MSO provision, including tele-education efforts, for more than 8 years, with progressive deterioration rather than improvement in the situation.

There is also the concern that obsolescence is already occurring with the advent of smartphones and tablets with access to innovative social media applications such as WhatsApp.^19^ The disadvantage of videoconferencing is that participants at each site have to be in a space which contains the appropriate equipment at an appointed time. This may be inconvenient for busy doctors, whether they are the specialists providing the tele-education or telemedicine, or the MOs at peripheral hospitals receiving the service. In contrast, the advantage of WhatsApp, for example, is that it can be used anywhere, provided mobile telephone reception is available, and at any time suited to the participants, perhaps while the MO is busy consulting a patient where a specialist opinion is required.

The literature may be making the distinction between successful telehealth in high-income countries with reliable sophisticated technology, and the less achievable use of such technology in low- and middle-income countries where the MSO occurs mostly in rural or remote sites.^30,31^ Difficult decisions will need to be taken in the context of KZN and SA to ensure that no further fruitless expenditure of time and money is squandered on expensive technologies that may already have become outdated.

Reflections on the value of MSO within the wider health care system – and its alignment with the proposed NHI – are important. A core tenet of the NHI is its universal access to health care, including specialist care. Hospital-based MSO improves access to specialist services and therefore its omission, apart from a cursory mention of ‘service outreach’, as a strategy in the NHI is of great concern.^20^ Hospital-based MSO strengthens the referral and support structures of the district health system, and yet the NHI White Paper of 2016 only discussed DCST. This is a very different type of district-based outreach with administrative rather than clinical responsibilities, which, as well as criticism from participants in this study, has received both queries and support in the SA medical literature.^21,33,34^ The intention is that DCST should complement MSO, but the danger is that of duplication. The problem is that SA does not have enough specialists in the public sector to populate either group fully, let alone both. An illustration of this is that very few of the 51 DCSTs countrywide have complete teams.^34^

To enable their implementation and sustainability, MSO programmes require a suitable structure and dedicated resource allocation for posts, transport and administrative support, in addition to the development of all-important longstanding relationships. The tension between a one-size-fits-all policy and local responsiveness is evident in the reflections above, showing the underlying contradiction between the need for central standardisation and flexible arrangements adapted to serve the local situation.

There is a vital research question requiring prompt elucidation in SA: what proportion of each specialist’s time should be allocated to MSO? The answer to this may show the way forward.

### Limitations of the study

Although this study is authentic, reflecting actual happenings rather than an artificial environment, a limitation is that it is descriptive, reporting and reflecting on the perceptions of the clinicians involved in MSO. It could not go into a deeper analysis of the direct benefit of the MSO to the individual patients or the relevant hospitals, which is an urgently required research. The study is also geographically specific to the PMB catchment area of western KZN and therefore, while the descriptions may echo dynamics in similar settings, the findings cannot necessarily be generalised. Nevertheless, it is likely that the findings are applicable to similar outreach contexts.

Although the study gives a wide freely expressed opinion from doctors familiar with giving or receiving MSO, the wealth of information is also a potential weakness, as it could not possibly all be used in a single publication. Therefore, the selection of relevant interview materials requires considerable attention to objectivity so that rigour could be maintained.

## Conclusion

Seven dominant themes describe enabling factors for an MSO programme. Long-term relationships are crucial to MSO. The structure of MSO implies both uniformity and flexibility. Effective MSO requires regularity. Available data are often not properly documented or utilised. Transport is critical to the existence of MSO. Telehealth is desirable, but technology failure and potential obsolescence jeopardise it. Multifaceted specialist outreach should be part of NHI policy.
